# Impact of Previous ART and of ART Initiation on Outcome of HIV-Associated Tuberculosis

**DOI:** 10.1155/2012/931325

**Published:** 2012-03-22

**Authors:** Enrico Girardi, Fabrizio Palmieri, Claudio Angeletti, Paola Vanacore, Alberto Matteelli, Andrea Gori, Sergio Carbonara, Giuseppe Ippolito

**Affiliations:** ^1^Clinical Epidemiology Unit, Department of Epidemiology and Clinical Department, National Institute for Infectious Diseases L. Spallanzani, Via Portuense 292, 00149 Rome, Italy; ^2^Institute of Infectious and Tropical Diseases, University of Brescia, 25123 Brescia, Italy; ^3^Division of Infectious Diseases, Department of Internal Medicine, “San Gerardo” Hospital, University of Milano-Bicocca, 20900 Monza, Italy; ^4^Clinic of Infectious Diseases, University of Bari, 70124 Bari, Italy

## Abstract

*Background*. Combination antiretroviral therapy (cART) has progressively decreased mortality of HIV-associated tuberculosis .To date, however, limited data on tuberculosis treatment outcomes among coinfected patients who are not ART-naive at the time of tuberculosis diagnosis are available. *Methods*. A multicenter, observational study enrolled 246 HIV-infected patients diagnosed with tuberculosis, in 96 Italian infectious diseases hospital units, who started tuberculosis treatment. A polytomous logistic regression model was used to identify baseline factors associated with the outcome. A Poisson regression model was used to explain the effect of ART during tuberculosis treatment on mortality, as a time-varying covariate, adjusting for baseline characteristics. *Results*. Outcomes of tuberculosis treatment were as follows: 130 (52.8%) were successfully treated, 36 (14.6%) patients died in a median time of 2 months (range: 0–16), and 80 (32.6%) had an unsuccessful outcome. Being foreign born or injecting drug users was associated with unsuccessful outcomes. In multivariable Poisson regression, cART during tuberculosis treatment decreased the risk of death, while this risk increased for those who were not ART-naive at tuberculosis diagnosis. *Conclusions*. ART during tuberculosis treatment is associated with a substantial reduction of death rate among HIV-infected patients. However, patients who are not ART-naive when they develop tuberculosis remain at elevated risk of death.

## 1. Introduction

The wider access to combined antiretroviral therapy (cART) had a profound impact on HIV-associated tuberculosis.

Prospective studies conducted in high-burden and low-burden countries have clearly shown that incidence of tuberculosis is strikingly reduced in persons receiving cART [1–5], and more recently a randomized trial has shown that early initiation of cART was associated with a significant reduction of the incidence of extra pulmonary tuberculosis [6]. Nevertheless, incidence of tuberculosis may remain higher than that observed in non-HIV-infected persons even in cART treated patients [2, 7].

A number of observational studies have also demonstrated that starting cART during treatment for tuberculosis reduces mortality in ART-naive patients in spite of the increased risk of immune reconstitution syndrome [8]. The effect of early initiation of cART in HIV-infected patients with tuberculosis has been also confirmed in randomized clinical trials [9, 10].

However, little information is available on the outcome of tuberculosis occurring in patients who are not ART-naive at the time of tuberculosis diagnosis.

In a multicenter study conducted in Italy in the context of wide availability of cART, we found that more than one-third of cases of HIV-associated tuberculosis occurred in patients who were already on antiretroviral treatment [11]. In this paper we analyze the outcome of treatment of tuberculosis among patients enrolled in that study.

## 2. Patients and Methods

### 2.1. Study Design and Subjects Selection

Design of this multicenter, prospective, observational study has been previously described [11]. Briefly, 154 Italian infectious diseases hospital units were invited to participate in the study and 96 (62,3%) agreed to enroll patients. These units are located in 18 of Italy's 20 regions, and all of them are located in public hospital and specialize in HIV care.

Individuals (in and out-patients) 18 years of age or older, with confirmed HIV infection, diagnosed with tuberculosis in the participating units during a 15-month period, were included in the study. All patients were given an identification code to guarantee confidentiality and each of the participating centers sought ethical clearance according to local regulations.

### 2.2. Data Collection

At baseline, the following data were collected for each enrolled subject: age, sex, country of birth, place of residence, education, employment, date of first positive HIV test, mode of HIV infection, history of active tuberculosis, results of chest radiographs and clinical evaluation and microbiological examinations for mycobacterial infection and disease. Data on concomitant AIDS defining diseases, CD4+ lymphocyte count and current and/or previous antiretroviral therapy were also recorded. Followup data were obtained from hospital wards and outpatient clinic records, and included information on antiretroviral therapy, tuberculosis treatment outcome, and vital status at the end of the study period.

All data were collected onto coded standardised forms. All forms were checked by scientific staff at the coordinating center for logical errors.

Cultures for mycobateria were performed on radiometric method (BACTEC; Becton Dickinson, Microbiology Systems, Sparks, MD, USA) and/or Löwenstein-Jensen medium.

### 2.3. Definitions and Outcome Variables

A case of tuberculosis was defined as a physician diagnosis of tuberculosis in a person who has bacteriological evidence of active disease (isolation of *Mycobacterium tuberculosis* from a clinical specimen and/or demonstration of *M. tuberculosis* from a clinical specimen by nucleic acid amplification) and/or signs and symptoms compatible with tuberculosis (e.g., an abnormal, unstable chest radiographs, or clinical evidence of current disease), completed diagnostic evaluation and decision by physician to treat with a full course of antituberculosis chemotherapy. On the basis of microbiological, clinical, radiological or histological findings, tuberculosis was classified as pulmonary, both pulmonary and extra pulmonary, extra pulmonary only.

A new case of tuberculosis was defined as a patient who has never had treatment for tuberculosis, or who has taken antituberculosis drugs for less than one month. Previously treated cases were defined as patient who had received at least 1 month of antituberculous therapy in the past. Drug-resistant (DR) tuberculosis was defined as caused by a *M. tuberculosis* strain resistant to one or more first-line antituberculosis drugs but not to both isoniazid and rifampin, while multidrug-resistant (MDR) tuberculosis included cases resistant to at least both. Patients were defined aware of HIV serostatus if patients they had their first HIV positive test performed al least 3 months before tuberculosis diagnosis.

For the purpose of the present analysis, patients were defined ART-naive if they had never received antiretrovirals or they received antiretrovirals for less than one month before diagnosis of tuberculosis. Patients were defined on ART at the time of tuberculosis diagnosis if they received antiretrovirals for at least one month in the three months preceding tuberculosis diagnosis.

Tuberculosis treatment outcomes were defined according WHO definitions [12]. For the purpose of the analysis, outcomes were also grouped in successful outcome (including patients cured and with completed treatment) and unsuccessful outcome (including patients transferred out, defaulted and treatment failure).

### 2.4. Statistical Analysis

Descriptive statistical methods were used to provide a general profile of the study population. The *χ* ^2^ or Fisher's Exact Test, as appropriate, were used to compare proportions. Odds ratios (ORs) with the associated 95% confidence intervals (CI) were calculated to measure the association between variables and treatment outcome. By fitting a polytomous logistic regression, we analyzed association of baseline characteristics associated with death and unsuccessful outcome of tuberculosis treatment, compared to successful outcome.

To investigate the impact of cART on mortality rate, a Poisson regression model was used. Results of this analysis are presented as mortality rate ratios (MRRs) with the associated 95% CI. Patients were included from the initiation of antituberculosis treatment until completion of treatment, death or loss to followup, whichever comes first; cART was included in the analysis as a time-dependent variable together with potential confounders. Analyses were performed with STATA software (Stata Corp. Stata Statistical Software. College Station, TX, USA).

## 3. Results

### 3.1. Study Population

We considered for inclusion in the analysis 271 HIV-infected patients with who were diagnosed with tuberculosis during the study period. Among these patients, 25 (9.22%) did not start tuberculosis treatment, 5 because they were transferred-out and 20 who were lost to follow up immediately after diagnosis. The remaining 246 patients entered the present analysis.

Table 1 shows characteristics of patients at tuberculosis diagnosis. The majority of patients (80.2%) were males and the median age was 36.9 (range 21.27–76.03) years. Diagnosis of tuberculosis was confirmed by culture in 160 patients (74,7%); 125 patients had results of antimycobacterial drugs susceptibility testing, of whom 22 (17.6%) had drug-resistant tuberculosis and 4 (3.2%) multidrug-resistant tuberculosis. 

The median time from first date of documented HIV seropositivity was 36.9 months (range: 0–201.3), and 96 (39%) were not ART-naive at the time of tuberculosis diagnosis. Of these patients, 34 received antiretroviral therapy for a median of 13.5 months (range 1–86), but not in the three months preceding diagnosis of tuberculosis, and their last ART regimen included a protease inhibitor (PI) in 20 patients and a nonnucleoside reverse transcriptase inhibitor (NNRTI) in 11 patients. 

At baseline the median value of CD4 lymphocytes was 120.5/mmc (range 0–1111), and viral load median value, calculated in 241 patients, was 4.94 log copies/mL. At least one concomitant AIDS defining illness disease was recorded in 60 (24.4%) patients. 

### 3.2. Tuberculosis Treatment Outcome

We recorded tuberculosis treatment outcomes for the 246 patients included in the analysis. A successful outcome was recorded for 130 patients (52.8%), among them 75 (30.5%) were cured and 55 (22.4%) completed treatment. Eighty patients (32.5%) had unsuccessful outcomes: 44 (17.9%) were lost to follow up in a median time of 1 month, and 25 (10.2%) were defaulters, 9 (3.7%) were transferred-out, and 2 (0.8%) were failures. Thirty-six patients (14.6%) died a median time of 2 months after tuberculosis treatment initiation. 

Table 1 shows the distribution of treatment outcomes according to baseline patients' characteristics. 

In multivariable polytomous logistic regression analysis (Table 2), not being ART-naive was associated with an increase of the probability of unsuccessful outcomes. Being foreign born was associated with a threefold increase of the risk of unsuccessful outcomes (OR 3.38, 95% CI 1.38–8.29, *P* = 0.008), which was also more likely for injecting drug users. Risk of death was also associated with being injecting drug users as well as to a lower CD4 cells count at the time of tuberculosis diagnosis and MDR tuberculosis. 

### 3.3. Use of cART during Tuberculosis Treatment and Risk of Death

Among the patients enrolled, 151 (61.4%) received cART and tuberculosis treatment concurrently. Of these patients 62 were already on cART when tuberculosis was diagnosed and 89 started cART during tuberculosis treatment, 56 (62.9%) in the initial phase and 33 (37.1%) in the continuation phase and included a PI in. Patients who were already on cART at initiation of TB have been receiving antiretrovirals for a median of 24 months (range 3–108) before diagnosis of tuberculosis and their last cART regimen included a PI in 35 cases and an NNRTI in 23. An additional 21 patients were not ART-naive but not on ART at tuberculosis diagnosis. ART administered during tuberculosis treatment included a PI in 75 cases (49.7%). 

We performed a further analysis in order to estimate the impact of use of cART during tuberculosis treatment on death rate of HIV-infected patients with tuberculosis. 

During 161.2 person-years (PY) of observation, 36 deaths occurred with an overall mortality rate of 22.3 per 100 PY (95% CI: 16.1–31.0). Among the patients who died, 17 were not ART-naive, 7 were ART-naive and started cART during tuberculosis treatment and 12 patients never started cART. 

In multivariable analysis (Table 3), the use of cART during tuberculosis treatment significantly reduced the risk of death (IRR 0.14, 95% CI 0.06–0.30, *P* < 0.001), whereas being not ART-naive at tuberculosis diagnosis caused a more than four-fold increase in the same risk (IRR 4.04, 95% CI 1.09–14.96, *P* = 0.037). Risk of death was also associated with a lower CD4 cell count, age ≥ 40 at diagnosis, and MDR tuberculosis. 

## 4. Discussion

In this multicentre study conducted in a low tuberculosis incidence country, a successful outcome of tuberculosis treatment was documented in slightly more than 50% of HIV-infected patients; the death of the patient during treatment was recorded in almost 15% patients. When we analyzed the impact of cART before and during tuberculosis treatment on the risk of death, we found the cART use during tuberculosis treatment reduced the probability of dying, while this risk was increased in those who were not ART-naive at tuberculosis diagnosis. 

The overall success rate of tuberculosis treatment observed in the present study is lower than that reported in other European studies in general population. A survey conducted in 10 European countries [13] found an overall proportion of successful outcome of tuberculosis treatment of 69% with a range between 60% and 88% in different countries and a death rate of 1%. In a systematic review of European surveys [14], an overall success rate of 74.4% was recorded with a death rate of 6.9%. These discrepancies however were not unexpected. First of all a high proportion of patients in our study population were intravenous drug users (48%) or foreign born (34%), and both these characteristics have been associated with a greatly reduced probability of successful outcome of tuberculosis treatment. For example in a Spanish study [15] intravenous drug users and foreign born persons had a six-fold or higher increase of the risk of interrupting treatment. This association was observed also in our analysis, in which the death outcome was also more likely for intravenous drug users. 

However, the main difference with surveys of tuberculosis treatment outcome in general population is the increased proportion of death, and HIV infection per se may most likely account for the observed discrepancies in death rates. Indeed the above-referenced Spanish multicentre survey [15] found a six-fold increase in death rate among HIV-infected patients with tuberculosis compared to non-HIV infected patients. 

We further explored the association between the risk of death and the use of cART. In our study population, almost 50% of patients continued or started cART during the initiation phase of tuberculosis treatment and an additional 14% initiated cART during the continuation phase. Overall, the use of cART was associated with a greater than six-fold reduction of the risk of death. This effect is of the same order of magnitude of that observed both in high and low tuberculosis incidence countries [8]. Thus our data concur with available evidence suggesting the importance of starting cART early during tuberculosis treatment [16]. 

A relevant finding was the increased risk of death for individuals who were not ART-naive when diagnosed with tuberculosis, which remained significant when we adjusted in the analysis for current cART use as well as for other factors associated with the risk of death such as a low CD4 cells count, older age at tuberculosis diagnosis, and multidrug resistance. The reasons for this association remains to be elucidated, nevertheless some hypothesis can be put forward. First of all, some patients had interrupted cART treatment before tuberculosis was diagnosed and this may be a marker of poor adherence to cART or previous cART failure or cART toxicity. Thus, even if cART is resumed during tuberculosis treatment, a reduced effect could be expected in these patients. Moreover, treatment interruptions per se have been associated with an increased risk of death [17]. Occurrence of tuberculosis during cART may also be a marker of progression of HIV disease also when CD4 cell count and HIV viral load are taken into consideration, and indeed tuberculosis in cART-treated patients has been identified as an independent predictor of other HIV-associated clinical events and death [18]. 

Tuberculosis occurring in cART-treated patients may in some instances be due to the so-called unmasking, which is defined as clinical manifestation of preexisting tuberculosis infection that is due to ART-induced immune restoration and which may sometimes result in severe or even fatal disease [19]. We do not have clinical details to evaluate severity of tuberculosis in our patients. However, tuberculosis unmasking usually occurs during the first few weeks of cART [19, 20], while in the present study most of the patients who were not ART-naive were treated for several months before tuberculosis diagnosis. 

In this study we do not have details of antiretroviral treatment history of patients and in particular we cannot determine if those who were not ART-naive had virological treatment failures and/or antiretroviral resistance at the time of tuberculosis diagnosis, and thus we could not estimate the impact of these factors on patients' outcome. The high proportion of patients who abandoned treatment may also have affected the analysis of factors associated with death. A further limitation is that the study was conducted on patients treated relatively early in the cART era, and thus the conclusions on the effect on cART may not necessarily be applicable to the newer cART regimens. 

In conclusion these study shows an alarmingly high proportion of unsuccessful outcome of tuberculosis in HIV-infected persons who inject drugs or are migrants and stresses the need of intervention aimed at keeping these patients into care. 

Tuberculosis occurred frequently in patients who were not ART-naive, and these patients had an increased risk of death compared to those who were ART-naive, also after taking into account the use of ART during tuberculosis treatment. Tuberculosis occurring in patients who already received ART may represent in the future an important issue to be addressed in high tuberculosis incidence countries in which scaling up of ART is currently underway. 

## Figures and Tables

**Table 1:** Characteristics of 246 HIV-infected patients at tuberculosis diagnosis and outcome of treatment.

Variable	*n* (%)	Successful outcome *n* (%)	Death *n* (%)	Unsuccessful outcome *n* (%)	*P* value
Sex					
Male	199	97 (48.7)	32 (16.1)	70 (35.2)	0.035
Female	47	33 (70.2)	4 (8.5)	10 (21.3)	
Place of origin					
Born in Italy	162	84 (51.9)	29 (17.9)	49 (30.2)	0.11
Foreign born	84	46 (54.8)	7 (8.3)	31 (36.9)	
Age at TB diagnosis (years)					
<40	162	87 (53.7)	18 (11.1)	57 (35.2)	0.084
≥40	84	43 (51.2)	18 (21.4)	23 (27.4)	
Education (years)					
0–8	146	75 (51.4)	21 (14.4)	50 (34.2)	0.850
9–18	51	26 (51.0)	9 (17.6)	16 (31.4)	
Unknown	49	29 (59.2)	6 (12.2)	14 (28.6)	
History of imprisonment					
Yes	24	12 (50.0)	4 (16.7)	8 (33.3)	0.91
No/unknown	222	118 (53.2)	32 (14.4)	72 (32.4)	
Mode of HIV infection					
Injecting drug use	119	49 (41.2)	25 (21.0)	45 (37.8)	0.001
Other	127	81 (63.8)	11 (8.7)	35 (27.6)	
Housing					
Private	209	116 (55.5)	33 (15.8)	60 (28.7)	0.014
Community/homeless	37	14 (37.8)	3 (8.1)	20 (54.1)	
CD4 lymphocyte count (cells/mmc)					
0–199	156	81 (51.9)	26 (16.7)	49 (31.4)	0.18
200–350	42	20 (47.6)	8 (19.0)	14 (33.3)	
>350	48	29 (60.4)	2 (4.2)	17 (35.4)	
AIDS					
Yes	132	65 (49.2)	27 (20.5)	40 (30.3)	0.019
No	114	65 (57.0)	9 (7.9)	40 (35.1)	
Site of diseases					
Pulmonary	197	101 (51.3)	28 (14.2)	68 (34.5)	0.413
Extra pulmonary	49	29 (59.2)	8 (16.3)	12 (24.5)	
History of TB treatment					
New cases	206	113 (54.9)	28 (13.6)	65 (31.6)	0.279
Previously treated cases	40	17 (42.5)	8 (20.0)	15 (37.5)	
Drug susceptibility test^#^					
Susceptible TB	99	54 (54.5)	15 (15.2)	30 (30.3)	0.387
DRTB	22	13 (59.1)	2 (9.1)	7 (31.8)	
MDR TB	4	1 (25.0)	2 (50.0)	1 (25.0)	
Awareness of HIV seropositivity					
Yes	176	79 (44.9)	31 (17.6)	66 (37.5)	<0.001
No	70	51 (72.9)	5 (7.1)	14 (20.0)	
cART naive					
Yes	150	84 (56.0)	16 (10.7)	50 (33.3)	0.091
No	96	46 (47.9)	20 (20.8)	30 (31.2)	
Concomitant diseases at TB diagnosis					
Yes	60	30 (50.0)	15 (25.0)	15 (25.0)	0.033
No	186	100 (53.8)	21 (11.3)	65 (34.9)	

^#^Calculated on 125 patients with drug susceptibility test performed.

**Table 2:** Multivariable odds ratios* of unsuccessful outcome of tuberculosis treatment and death according to baseline characteristics for 246 HIV-infected patients with tuberculosis.

Variable	Unsatisfactory outcome of TB treatment		Death	
	OR (95% CI)	*P* value	OR (95% CI)	*P* value
Sex				
Male	1.00		1.00	
Female	0.38 (0.16–0.90)	0.029	0.35 (0.10–1.27)	0.112
Place of origin				
Born in Italy	1.00		1.00	
Foreign born	3.66 (1.48–9.06)	0.005	1.98 (0.52–7.45)	0.314
Age at TB diagnosis (years)				
<40	1.00		1.00	
≥40	1.04 (0.52–2.11)	0.909	2.34 (0.94–5.84)	0.068
Education (years)				
0–8	1.00		1.00	
9–18	1.00 (0.44–2.27)	0.996	0.81 (0.27–2.37)	0.698
Unknown	0.83 (0.35–1.96)	0.671	0.69 (0.20–2.35)	0.550
History of imprisonment				
Yes	1.00		1.00	
No/unknown	1.93 (0.65–5.74)	0.238	2.17 (0.53–8.90)	0.282
Mode of HIV infection				
Injecting drug use	1.00		1.00	
Other	0.42 (0.19–0.93)	0.033	0.25 (0.08–0.74)	0.012
Housing				
Private	1.00		1.00	
Community/homeless	2.21 (0.93–5.30)	0.074	0.44 (0.09–2.30)	0.333
CD4 lymphocyte count (increase: 50 cells)	0.96 (0.88–1.04)	0.326	0.83 (0.71–0.97)	0.020
AIDS				
Yes	1.00		1.00	
No	0.90 (0.45–1.82)	0.778	0.44 (0.16–1.19)	0.106
Site of diseases				
Pulmonary	1.00		1.00	
Extra pulmonary	0.66 (0.29–1.50)	0.320	1.15 (0.42–3.18)	0.782
History of TB treatment				
New cases	1.00		1.00	
Previously treated cases	0.96 (0.39–2.36)	0.930	0.71 (0.22–2.32)	0.572
Drug susceptibility test				
Susceptible TB	1.00		1.00	
DR TB	1.03 (0.33–3.21)	0.962	0.44 (0.07–2.69)	0.376
MDR TB	0.83 (0.03–20.52)	0.911	37.10 (1.98–693.79)	0.016
Not available	1.41 (0.71–2.78)	0.322	1.24 (0.50–3.07)	0.649
Awareness of HIV seropositivity and HIV treatment history				
Not aware	1.00		1.00	
Aware and cART naive	5.11 (2.04–12.80)	0.001	2.95 (0.77–11.33)	0.115
Not cART naive	3.31 (1.29–8.46)	0.013	4.04 (1.09–14.96)	0.037
Concomitant diseases at TB diagnosis				
Yes	1.00		1.00	
No	1.38 (0.59–3.26)	0.461	0.59 (0.23–1.55)	0.286

*From polytomous logistic regression model with successful outcome as reference category.

**Table 3:** Univariable and multivariable mortality rate ratio (MRR)* for 246 HIV-infected patients with tuberculosis.

Variable	Univariable analysis		Multivariable analysis	
	MRR (95% CI)	*P* value	MRR (95% CI)	*P* value
Sex				
Male	1.00		1.00	
Female	0.52 (0.18–1.47)	0.217	0.46 (0.15–1.38)	0.163
Place of origin				
Born in Italy	1.00		1.00	
Foreign born	0.50 (0.22–1.15)	0.102	1.36 (0.42–4.41)	0.608
Age at TB diagnosis				
<40	1.00		1.00	
≥40	1.96 (1.02–3.77)	0.043	2.73 (1.26–5.93)	0.011
Education (years)				
0–8	1.00		1.00	
9–18	1.14 (0.52–2.50)	0.736	0.92 (0.39–2.21)	0.855
Unknown	0.81 (0.33–2.02)	0.659	1.00 (0.36–2.76)	0.999
History of imprisonment				
Yes	1.00		1.00	
No/unknown	0.80 (0.28–2.26)	0.675	2.05 (0.64–6.60)	0.282
Mode of HIV infection				
IDU	1.00		1.00	
Other	0.37 (0.18–0.75)	0.006	0.39 (0.15–0.99)	0.047
Housing				
Private	1.00		1.00	
Community/homeless	0.71 (0.22–2.32)	0.573	0.29 (0.07–1.17)	0.082
CD4 lymphocyte count (per 50 cells increase)	0.85 (0.75–0.96)	0.010	0.83 (0.72–0.95)	0.006
AIDS				
Yes	1.00		1.00	
No	0.40 (0.19–0.85)	0.017	0.47 (0.20–1.13)	0.091
Site of diseases				
Pulmonary	1.00		1.00	
Extra pulmonary	0.94 (0.43–2.07)	0.888	1.36 (0.59–3.11)	0.471
History of TB treatment				
New cases	1.00		1.00	
Previously treated cases	1.53 (0.70–3.35)	0.292	0.78 (0.32–1.94)	0.597
Drug susceptibility test				
Susceptible TB	1.00		1.00	
DR TB	0.48 (0.11–2.12)	0.336	0.34 (0.07–1.68)	0.184
MDR TB	3.91 (0.90–17.12)	0.070	35.50 (5.50–229.19)	<0.001
Not available	0.85 (0.43–1.71)	0.658	1.34 (0.60–3.00)	0.471
Awareness of HIV seropositivity and HIV treatment history				
Not aware	1.00		1.00	
Aware and ART-naive	2.64 (0.92–7.60)	0.072	1.73 (0.52–5.72)	0.370
Not ART-naive	3.41 (1.28–9.08)	0.014	4.27 (1.27–14.29)	0.019
cART (time dependent)				
No	1.00		1.00	
Yes	0.26 (0.13–0.52)	<0.001	0.14 (0.06–0.30)	<0.001
Concomitant diseases at TB diagnosis				
Yes	1.00		1.00	
No	0.43 (0.22–0.83)	0.012	0.63 (0.29–1.35)	0.231

*Estimated by a Poisson regression model.

## References

[1] Badri M, Wilson D, Wood R (2002). Effect of highly active antiretroviral therapy on incidence of tuberculosis in South Africa: a cohort study. *The Lancet*.

[2] Lawn SD, Badri M, Wood R (2005). Tuberculosis among HIV-infected patients receiving HAART: long term incidence and risk factors in a South African cohort. *AIDS*.

[3] Lawn SD, Myer L, Edwards D, Bekker LG, Wood R (2009). Short-term and long-term risk of tuberculosis associated with CD4 cell recovery during antiretroviral therapy in South Africa. *AIDS*.

[4] Girardi E, Antonucci G, Vanacore P (2000). Impact of combination antiretroviral therapy on the risk of tuberculosis among persons with HIV infection. *AIDS*.

[5] Santoro-Lopes G, De Pinho AMF, Harrison LH, Schechter M (2002). Reduced risk of tuberculosis among Brazilian patients with advanced human immunodeficiency virus infection treated with highly active antiretroviral therapy. *Clinical Infectious Diseases*.

[6] Cohen MS, Chen YQ, McCauley M (2011). Prevention of HIV-1 infection with early antiretroviral therapy. *The New England Journal of Medicine*.

[7] Girardi E, Sabin CA, Monferte AD (2005). Incidence of tuberculosis among HIV-infected patients receiving highly active antiretroviral therapy in Europe and North America. *Clinical Infectious Diseases*.

[8] Kwan C, Ernst JD (2011). HIV and tuberculosis: a deadly human syndemic. *Clinical Microbiology Reviews*.

[9] Abdool Karim SS, Naidoo K, Grobler A (2010). Timing of initiation of antiretroviral drugs during tuberculosis therapy. *The New England Journal of Medicine*.

[10] Blanc F-X Significant enhancement in survival with early (2 weeks) vs. late (8 weeks) initiation of highly active antiretroviral treatment (HAART) in severely immunosuppressed HIV-infected adults with newly diagnosed tuberculosis.

[11] Girardi E, Antonucci G, Vanacore P (2004). Tuberculosis in HIV-infected persons in the context of wide availability of highly active antiretroviral therapy. *European Respiratory Journal*.

[12] World Health Organization *Treatment of Tuberculosis. Guidelines for National Programmes*.

[13] Falzon D, Le Strat Y, Belghiti F, Infuso A (2005). Exploring the determinants of treatment success for tuberculosis cases in Europe. *International Journal of Tuberculosis and Lung Disease*.

[14] Faustini A, Hall AJ, Perucci CA (2005). Tuberculosis treatment outcomes in Europe: a systematic review. *European Respiratory Journal*.

[15] Caylà JA, Caminero JA, Rey R, Lara N, Vallés X, Galdós-Tangüis H (2004). Current status of treatment completion and fatality among tuberculosis patients in Spain. *International Journal of Tuberculosis and Lung Disease*.

[16] World Health Organization (2009). *Treatment of Tuberculosis: Guidelines*.

[17] Kaufmann GR, Elzi L, Weber R (2011). Interruptions of cART limits CD4 T-cell recovery and increases the risk for opportunistic complications and death. *AIDS*.

[18] Komati S, Shaw PA, Stubbs N (2010). Tuberculosis risk factors and mortality for HIV-infected persons receiving antiretroviral therapy in South Africa. *AIDS*.

[19] Meintjes G, Rabie H, Wilkinson RJ, Cotton MF (2009). Tuberculosis-associated Immune Reconstitution Inflammatory Syndrome and Unmasking of Tuberculosis by Antiretroviral Therapy. *Clinics in Chest Medicine*.

[20] Manabe YC, Breen R, Perti T,  Girardi E, Sterling TR (2009). Unmasked tuberculosis: part of a disease spectrum after highly active antiretroviral therapy. *Journal of Infectious Diseases*.

